# Regenerative Endodontic Treatment of an Infected Immature Dens Invaginatus with the Aid of Cone-Beam Computed Tomography

**DOI:** 10.1155/2014/403045

**Published:** 2014-10-30

**Authors:** Işıl Kaya-Büyükbayram, Şerife Özalp, Emre Aytugar, Seda Aydemir

**Affiliations:** ^1^Acibadem Hospital Endodontics, Istanbul, Turkey; ^2^Department of Pediatrics, Faculty of Dentistry, Bezmialem University, Istanbul, Turkey; ^3^Department of Oral Diagnosis and Radiology, Faculty of Dentistry, Bezmialem University, Istanbul, Turkey; ^4^Department of Endodontics, Faculty of Dentistry, Kocaeli University, Kocaeli, Turkey

## Abstract

Dens invaginatus is a developmental anomaly that results in an enamel-lined cavity intruding into the crown or root before the mineralization phase. This report presents regenerative endodontic treatment of a necrotic immature tooth with Oehler's type III dens invaginatus of a nine-year-old female patient. A diagnosis of dens invaginatus (Oehler's type III) and a large periapical lesion was established with the aid of cone-beam computed tomography (CBCT). In the presented case contrary to the classic revascularization protocol, mechanical instrumentation was performed which apparently did not interfere with the regeneration process. After mechanical instrumentation of the invaginated canal by manual K-files, the invaginated canal space was disinfected by triple antibiotic paste followed by blood clot induction from the periapical tissues and the placement of mineral trioxide aggregate. At one-year follow-up, the tooth remained clinically asymptomatic. Radiographic examination revealed complete healing of the periapical lesion. At the 20-month follow-up, the radiographic examination also showed that the open apex was closed and the walls of the root canal were thickened.

## 1. Introduction

Dens invaginatus, known more commonly as “dens in dente,” is an unusual developmental anomaly resulting in a deepening or invagination of the enamel organ into the dental papilla before the mineralization phase. The etiology of dens invaginatus is still unclear and several theories have been suggested, including alterations in tissue pressure, trauma, infection, or local discrepancy in the cellular hyperplasia [1].

In 1953, Hallet was the first to document an attempt to classify dens invaginatus. He suggested the existence of four types of invagination based on both clinical and radiographic criteria [2]. A more widely used classification is that described by Oehlers. In his system, Oehlers categorizes invaginations into three classes, based on how far they extend radiographically from the crown into the root. Type I represents an enamel invagination confined within the tooth crown, type II is an enamel-lined form that invades the root as a blind sac and may communicate with the pulp, and type III is a severe form where the invagination extends beyond the cementoenamel junction and exhibiting a second foramen into the lateral periodontal ligament or the periapical tissue [3].

The incidence of dens invaginatus has been reported to be in the range of 0.04%–10%, with the upper lateral incisors most commonly involved [4].

Endodontic treatment of dens invaginatus type III can become complicated because of an unpredictable internal anatomy [1]. A complete disinfection of the root canal system is of great importance to promote healing of periapical tissues. Recently, the advent of cone-beam computed tomography (CBCT), a three-dimensional (3D) imaging modality, has helped in diagnosis and better treatment planning of such complex cases requiring endodontic therapy [5, 6]. One of the major problems in endodontic therapy in teeth with pulp necrosis and open apex is obtaining an adequate closure of the root canal system. Regeneration treatment is a biologically based alternative approach to treat necrotic immature teeth. This treatment, unlike the apexification and artificial barrier technique, allows for the continuation of root development [7]. This approach is based on the occurrence of osteo/odontoprogenitor stem cells in the apical papilla that are resistant to the infection and necrosis caused by proximity to periodontal blood supply [8]. With this treatment, the objective is to create an appropriate environment inside the root canal space, including the absence of bacteria and necrotic pulp tissue and the presence of a scaffold and a tight coronal seal. This would promote the repopulation of these stem cells, regeneration of pulp tissue, and continuation of root development [9].

In the early 1960s, Ostby, a pioneer of regenerative endodontic procedures, demonstrated that new vascularized tissue could be induced in the apical third of the root canal of endodontically treated mature teeth with necrotic pulps and apical lesions. This was achieved by the creation of a blood clot in the apical third of a cleaned and disinfected root canal by using an apically extended root canal file just before root canal filling [10].

In 2001, Iwaya et al. described a procedure which they termed revascularization. This was carried out on a necrotic immature mandibular second premolar with a chronic apical abscess. After a period of 30 months, they reported thickening of the root canal walls by mineralized tissue and continued root development [11].

In 2004, Banchs and Trope described a revascularization protocol. After accessing the root canal, they irrigated it with sodium hypochlorite (NaOCl) and chlorhexidine gluconate (CHX) and sealed it in a combination of 3 antibiotics in an attempt to disinfect it and stimulate periapical repair [12].

At present, the use of the term revascularization is debatable. Trope [13] claimed that the term revascularization was chosen because the nature of tissue formed posttreatment was unpredictable and the only certainty was the presence of a blood supply; hence, it was revascularized.

Lenzi and Trope [14] suggested the term revitalization be used as a more appropriate term, as it is descriptive of the nonspecific vital tissue that forms in the root canal. In 2008, Hargreaves et al. [9] challenged the use of the term revascularization for regenerative endodontic procedures, claiming that the goal of treatment was to generate a pulp-dentin complex with functional properties that are capable of supporting continued root development while resolving apical periodontitis.

In our perspective regeneration, the term indicates an overall goal of reproducing the original tissue histology and function and the current protocol should be revised to attain a more biocompatible strategy. More importantly, the effect of adding tissue engineering triad components (stem cells, bioactive scaffolds, and growth factors) to the current protocol needs to be further researched [15]. Hopefully, universally accepted terms for these procedures will eventually be considered and resolved by the American Association of Endodontics.

The purpose of this report is to present a clinical case in which regenerative endodontic treatment was applied as a conservative method to successfully treat a maxillary lateral incisor with type III dens invaginatus and a large periapical lesion.

## 2. Case Presentation

**Figure 1:**
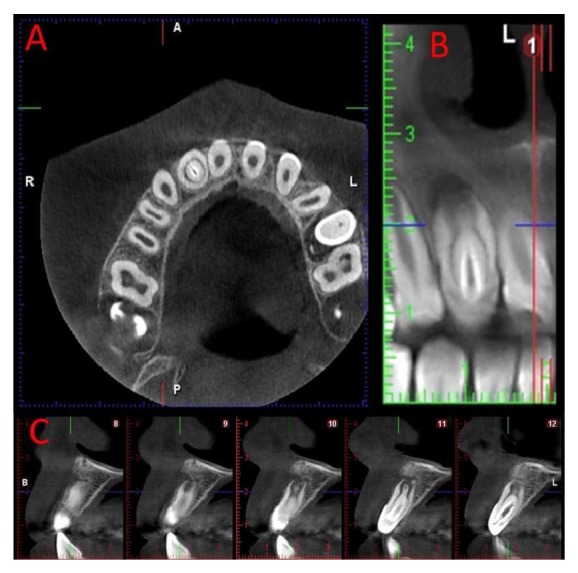
Three-dimensional image taken with CBCT.

A 9-year-old female patient was referred to Bakirkoy Acibadem Hospital with a complaint of pain on her maxillary right lateral incisor. She suffered from mild swelling associated with the tooth. Clinically, the crown of the maxillary right lateral incisor was cone-shaped and without caries. The tooth was sensitive to percussion. The periodontal status was normal (probing depth < 3 mm around the tooth). The tooth showed no mobility. The initial periapical radiographic examination revealed that the tooth showed an invagination (Oehlers' type III) and a large periapical lesion. The tooth had a diagnosis of symptomatic apical periodontitis. A CBCT scan was taken to see a three-dimensional image of this complex anatomy (Figure 1). The tooth showed a positive response to electric pulp vitality test.

Regenerative endodontic treatment was recommended. After reviewing the risks, benefits, and treatment options with her parents, an informed assent and consent were obtained for performing the recommended procedure. Under local anaesthesia, rubber dam was placed and endodontic access was performed. A single canal orifice was exposed. The vital pulp tissue was seen in the invaginated canal but access to the main root canal was not possible. Because the invaginated canal was thin, it was instrumented up to a size 30 K-file (Dentsply-Maillefer) and irrigated with 2.5% sodium hypochlorite (NaOCl) solution. Calcium hydroxide (Ca(OH)_2_) (Sultan Chemists Inc., Englewood, NJ, USA) was then applied into the root canal.

Although the above-mentioned treatment was applied, the tooth was still symptomatic after three weeks. The buccal gingiva was swollen. So, as a further step, triantibiotic paste (a creamy paste made of equal proportions of ciprofloxacin, metronidazole, and minocycline mixed with sterile water) was placed into the canal. One month after the antibiotic treatment, the tooth was asymptomatic.

In the next phase of the treatment, the regeneration protocol was performed. Firstly, the triantibiotic paste was removed from the canal by irrigating the invaginated canal with 2.5% NaOCl and saline solution. Afterwards, the canal was dried with paper points. The irritation of the periapical tissues beyond the apex of the invaginated canal was performed using a size 30 K file. Five minutes after bleeding started, and a mineral trioxide aggregate (MTA) (MTA-A; Angelus, Londrina, Brazil), plug was placed over the blood clot. A moist sterile cotton pellet was placed over the MTA and the access was sealed with a temporary restoration Cavit G (3 M ESPE Dental-Medizin GmbH Co, Seefeld, Germany). At the next appointment, two weeks later Fuji IX glass ionomer cement (Fuji Corporation, Osaka, Japan) and composite (Filtek Z250; 3 M ESPE) were used in order to perform coronal restoration.

**Figure 2:**
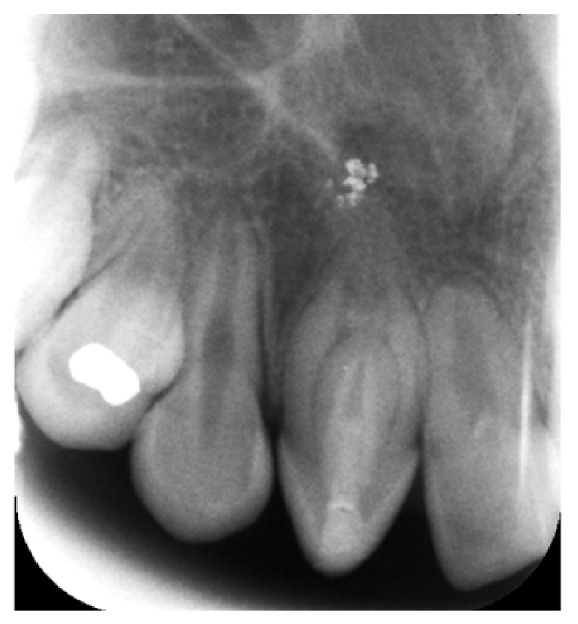
The one-year follow-up radiograph showing complete healing of the periapical lesion.

**Figure 3:**
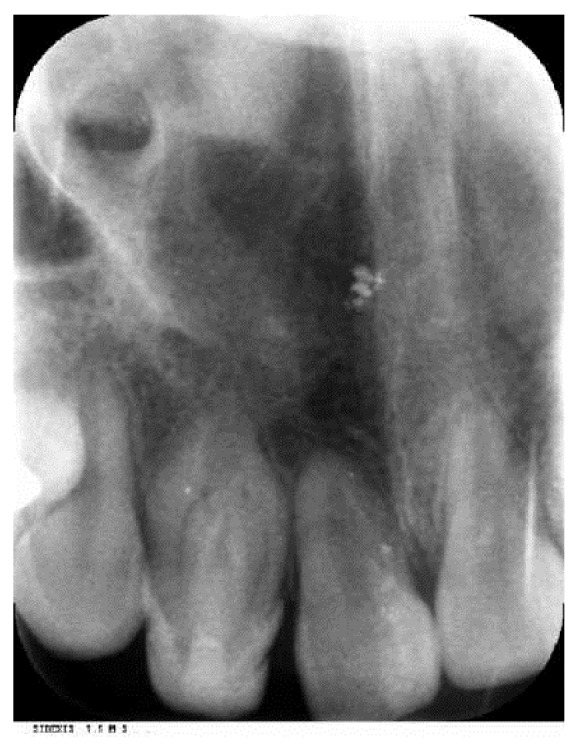
The twenty-month follow-up radiograph showing that the open apex was closed.

The one-year follow-up showed no clinical signs of pathology. Radiographic examination revealed complete healing of the periapical lesion, but apical closure was not observed (Figure 2). The tooth had a negative response to vitality testing. The vital pulp tissue in the invaginated canal had been removed during instrumentation; however, the main root canal was not exposed, the tooth had a negative response to vitality testing postoperatively, and the periapical lesion resolved. So we assume that it was a peri-invagination lesion. At the twenty-month examination, the control radiograph showed successful apical closure (Figure 3). At this follow-up examination, the tooth had a negative response to vitality testing.

## 3. Discussion

The treatment of an immature permanent tooth with periapical pathosis is a challenge in paediatric dentistry, especially in the case of dens invaginatus. The various treatment options include apexification [16], nonsurgical endodontic treatment [17], a combination of nonsurgical and surgical endodontic treatment [5], obturation of the invagination alone while maintaining pulp vitality [18], and the removal of the dens invaginatus from the root canal [19].

In the presented case, there was a large periapical lesion in the immature tooth with type III dens invaginatus. To promote root development, endodontic treatment should be performed. Apexification is the traditional treatment for necrotic teeth with open apices [16], but in this case, Ca(OH)_2_ medication was not sufficient to eliminate the infection, so traditional apexification treatment was not suitable for the presented case.

Other nonsurgical endodontic treatment procedures are the complete elimination of the invagination with 70 or larger K files [20] or the removal of the dens invaginatus from the root canal [19]. In both techniques, however, the root becomes weaker and is more likely to fracture.

In recent years, several case reports have showed that continued root development and complete periapical healing can be achieved with the treatment of an immature permanent tooth with pulp necrosis by a regenerative technique [21, 22]. Because regenerative endodontics is a conservative method leading the apex to close while the root canal walls become thicker, it prevents the root from fracturing and reaching long-term retention. Therefore, pulp regeneration is the ideal treatment for the presented case.

Contrary to most of the published articles about classic revascularization protocol, in this case, mechanical instrumentation was performed in the invaginated canal to facilitate the insertion of intracanal medication, which apparently did not interfere with the regeneration process. In regeneration treatment, studies have reported different methods for disinfecting the necrotic immature tooth, including the use of triple antibiotic paste [21, 22] or calcium hydroxide [23, 24]. First, calcium hydroxide intracanal medication was applied for three weeks to decontaminate the root canal, which did not promote coronal discoloration [24]; however, the infection was not under control, so triantibiotic paste was used as a second choice.

**Figure 4:**
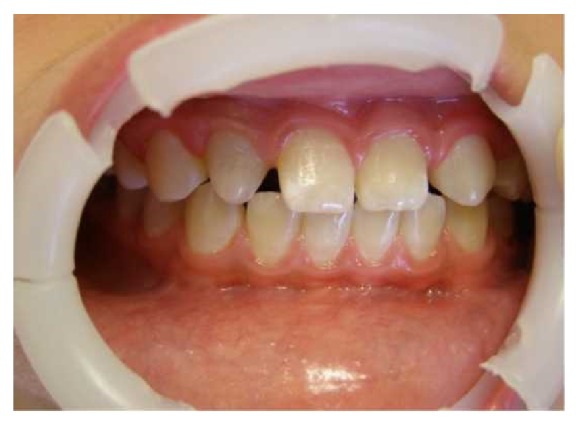
Clinical photography of maxillary right lateral incisor at the twenty-month follow-up appointment.

Radiographic follow-up after 20 months showed that the open apex had closed, while the walls of the root canal became thicker. Antibiotic paste and MTA caused the discoloration of the tooth (Figure 4). At this follow-up examination, the tooth had a negative response to vitality testing. The negative response to vitality testing may be because of the absence of vital pulp or pulp-like tissue or because the tissue in the canal space was probably not innervated or because of the presence of MTA sealing.

This report of pulp regeneration shows that the mechanical instrumentation of the invaginated canal in addition to intracanal medication with triple antibiotic paste led to satisfactory root development in a type III immature dens invaginatus with a large periapical lesion.
